# Targeting inhibitory control in youth irritability: challenges and opportunities

**DOI:** 10.1038/s41386-025-02151-x

**Published:** 2025-07-01

**Authors:** Simone P. Haller, Parmis Khosravi, Katherine Y. Kim, Melissa A. Brotman

**Affiliations:** Emotion and Development Branch, National Institute of Mental Health, Bethesda, MD USA

**Keywords:** Human behaviour, Cognitive control

## Abstract

Pediatric irritability, an increased proneness to anger relative to same aged peers, is linked to deficits in inhibitory control. Here, we examine issues of measurement and specificity of the construct of inhibitory control, with a focus on potential treatment implications. We cover lessons learned from prior training approaches. We dissect the role of inhibitory control in an efficacious psychosocial treatment and propose a research agenda to further test inhibitory control as a candidate behavioral treatment target for youth with irritability.

Inhibitory control, defined as the ability to override or suppress a dominant internal predisposition or external pull, is critical to adaptive functioning and an important tool through which individuals regulate emotional reactivity and associated ill-serving or inappropriate behavioral responses [1]. Inhibitory control is part of a set of high-level processes bundled under the construct of executive functions. Pediatric irritability, one of the most common presenting problems requiring clinical care in youth [2], is defined as a proneness to anger that manifest as temper outbursts and a persistently angry mood [3]. Clinically, temper outbursts can be conceptualized as an inability to inhibit a behavioral response in a negative affective state (e.g., yelling, slamming a door, throwing an object), behaviors that are relatively normative during preschools years, but decrease with age, parallelling protracted maturation in children’s ability to engage in inhibitory control behavior [4]. Epidemiological studies suggest impairing irritability presents in 2–5% of youth [5]. While over time some manifestations of irritability naturally decrease with development, in others, sustained irritability during childhood and adolescence requires intervention [6, 7]. In this perspective piece, we explore issues of construct measurement and specificity of inhibitory control. We discuss lessons learned from prior approaches of targeting inhibitory control in related phenotypes. We end by discussing the potential role of inhibitory control in exposure-based cognitive behavioral therapy and propose a research agenda to assess inhibitory control as a mediator of improvement.

## Issues of measurement and specificity

**Fig. 1:**
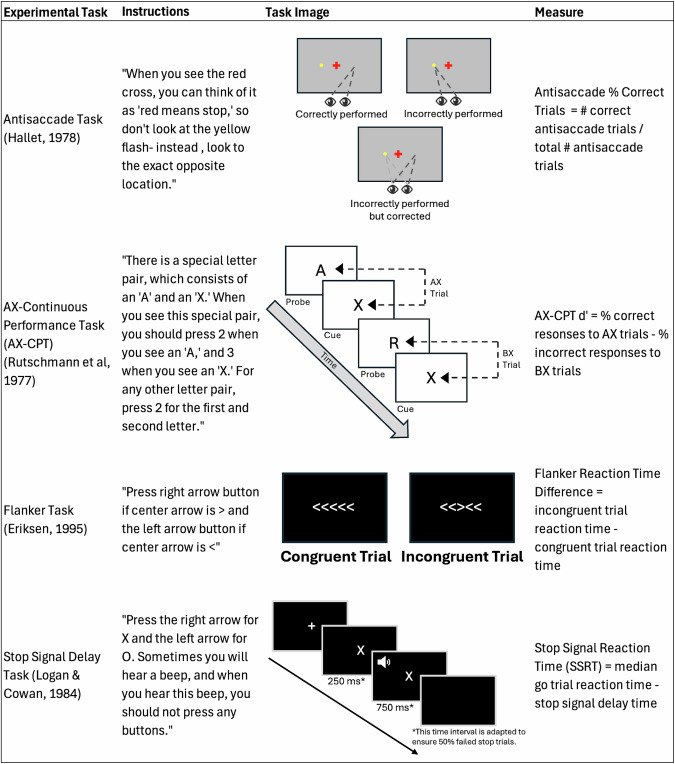
Illustration of four canonical inhibitory control tasks and commonly extracted metrics.

From a treatment target perspective, issues of measurement and specificity plague the construct of inhibitory control. Correlations amongst differently sourced measures of inhibitory control (e.g., self-report, behavioral probes) are often weak; indices, specifically, behavioral probes, are often not sufficiently sensitive to capture broader clinical and developmental spectra (see Fig. 1 for an overview of behavioral probes) [8]. Measurement reliability, critical for the characterization of individual differences, while high for self-report surveys of inhibitory control, are highly variable or poor for behavioral tasks probing the same construct [9]. These are significant psychometric shortcomings, impeding progress in assessing inhibitory control as a potential treatment target. Data-driven statistical approaches can extract potentially more stable latent measures. However, a good model fit does not guarantee that these variables are valid indicators of the construct or sensitive to change over time. Reliability and validity are critical prerequisites for treatment measures.

An additional challenge is that latent variable models that aim to assess the cognitive structure of executive functions demonstrate that inhibitory control is not separable from a general factor, unlike other cognitive functions, which do separate (e.g., working memory, cognitive flexibility) [10]. While this general factor is psychometrically robust, it renders any specificity between associations of inhibitory control and specific clinical phenotypes difficult to understand from a causal perspective. It may therefore not be surprising that deficits in inhibitory control have emerged as a transdiagnostic vulnerability factor for multiple pediatric mood, anxiety, attention, and behavioral difficulties, rather than a dysfunction specific to a certain diagnosis or symptom dimension [11]. Irritability may be a signal for general risk for psychopathology: youth with irritability generally have higher rates of comorbid disorders, more functional impairment, and poorer treatment response compared to those without co-occurring symptoms of irritability [12]. Thus, inhibitory control may still be a promising treatment target, especially in cases chosen for their generalizability (i.e., those with multiple comorbidities, with the most common one being attention deficit/hyperactivity disorder –ADHD).

## A core issue in irritability?

Theoretical models suggest that impairments in inhibitory control mediate the behavioral expression of irritability, specifically the phasic aspect, i.e., temper tantrums [13]. Developmental [14–16] and individual difference studies examining youth with behavioral difficulties or irritability support the role of inhibitory control in modulating behavioral manifestations of temper outbursts [17, 18]. However, as irritability research is still in a relatively nascent state, very few studies have attempted to isolate the role or causally link inhibitory control deficits to temper outbursts. What if inhibitory control does not emerge as a core issue in irritability? Could it still be a promising avenue for treatment? There is a substantial motor inhibition component inherent in suppressing reactive, instrumental behavioral tendencies (e.g., talking back, yelling, throwing an object) in response to a frustrating event (e.g., being told to stop playing a video game). Long term successful inhibition of these motor actions should arguably, over time, reduce the reactivity these interactions elicit. In turn, the impulse to direct the anger outward in motor actions would not be reinforced, as occurs during extinction for instrumentally conditioned responses. Thus, even if motor inhibition does not specifically mediate the manifestation and recurrence of temper tantrums, directly addressing it in treatment may eventually ameliorate impairing symptoms.

## Targeting cognitive control in treatment

Non-pharmacological treatment strategies for pediatric irritability are needed; a toolbox of mechanism-informed behavioral treatments and technologically delivered, targeted cognitive trainings have the potential for profound public health impact. Critically, understanding the mechanisms by which a treatment impacts clinical phenomena is vital to modify treatments and improve outcomes.

Conceptually, training interventions that improve motor inhibition should help irritable youth inhibit frustration-driven motoric outbursts. Given the importance of inhibitory control and executive functions more broadly in development, a considerable number of approaches to improve general executive functions have been tested [19]. Most of the work in motor inhibition specifically has focused on youth with ADHD using computerized cognitive training. Results have been mixed—it remains unclear whether inhibitory control training leads to sustained, measurable behavioral improvements in daily life [20]. Near transfer describes gains on similar, but not identical, training tasks or contexts; far transfer describes gains maintained on markedly different tasks or contexts. While some studies have shown near transfer, successful far transfer has rarely been demonstrated [21]. For any intervention to have clinical efficacy, treatment-related gains must be generalizable beyond the training task or context.

Failure of transfer to new material and contexts could be due to several reasons. For inhibitory control trainings, it may be that trainings are too narrow a behavioral output to translate to untrained domains. Most of the commonly utilized behavioral tasks operationalize inhibitory control as suppressing a learned, repetitive motor response, usually a button-press in a “cold” setting, i.e., no affect is evoked prior to the task being performed, the task itself contains emotionally neutral stimuli, and the performance outcome is not motivationally relevant. From a treatment perspective, this is a narrow definition given the complex, clinically relevant behaviors and contexts, specifically the negative affective states, in which they occur. That is, learning how to hold back pressing a button during a computer task does not translate into to withholding broader movements during a lesson at school (e.g., putting down ball, playing with friends during recess) and initiating new behaviors (e.g., getting in line, walking inside).

Another possible explanation for limited transfer is that contextualized inhibitory control recruits the broader cognitive control system, not just domain-specific motor processes. In fact, inhibitory control in many instances may use significant working memory resources and require flexible switching. However, studies assessing the impact of broad executive function trainings with large samples and active control groups often report no training-related improvements on general cognitive abilities [22].

In summary, challenges and opportunities arise from therapeutically targeting a system in developmental flux, especially in youth developmentally lagging compared to their peers. Training focused solely on reactive response inhibition in artificial settings may narrowly strengthen motor control but fail to engage the neural networks needed for emotionally relevant inhibition. As a result, youth may learn to suppress a button press in a lab task but still struggle to inhibit a reactive verbal or physical outburst in a frustrating social interaction. However, it appears that programs that target more general cognitively demanding activities show little evidence for transfer to general cognitive ability [23]. Thus, it may be that successful intervention development for irritability may demand a domain-specific, yet integrated strategy to improve inhibitory control within an emotionally meaningful training setting, with room for a flexible and iterative approach that can adapt to changes in a dynamic environment and evolving abilities.

## Exposure-based cognitive behavioral therapy plus parent management training for irritability targets inhibitory control

Work by our group has shown preliminary efficacy for exposure-based cognitive behavioral therapy plus parent management training for irritability [24]. This treatment was developed from a translational model we developed [3] and expanded [13]. We articulate that youth with irritability have broad deficits in threat and reward processing. Many youths with irritability interpret ambiguous stimuli as more emotionally evocative and demonstrate increased sensitivity to rewards and non-reward receipt [25, 26]. We hypothesize that deficits in inhibitory control are common to both impaired reward and threat processing. From a temporal perspective, within an event (e.g., being asked to do a chore) that leads to irritability, there are two neurocognitive processes involved in immediate behavioral responses and sustained irritability. First, aberrant proximal inhibitory control leads to in the moment, maladaptive, motoric behavioral manifestations of frustration (e.g., stopping the chore). Second, more sustained environmental contingencies reinforce these maladaptive immediate responses (e.g., inconsistent reinforcement in the past for stopping the chore).

Arguably, exposure-based cognitive behavioral therapy plus parent management training improves both reactive and sustained, preparatory inhibitory control, respectively. During exposure-based cognitive behavioral therapy for irritability, the child is gradually and safely introduced to stimuli/situations (e.g., stopping video game play) that typically induce an angry response (e.g., yelling). By repeatedly experiencing the stimuli (e.g., stop videogame) and withholding the behavioral response (e.g., yelling), the child learns to tolerate and manage their anger by: (1) dissociate their “prepotent” typical response from the environmental stimuli, (2) withhold the typical motor response, and (3) engage in another goal-directed behavioral action pattern. Augmenting “in the moment” inhibitory control, parent management training teaches the caregiver consistent rules that reinforce specific goal-directed child responses (e.g., allowing the child continue videogame play following yelling vs. positively reinforcing stopping videogame play without behavioral or verbal outburst). As the child’s participation is critical in this treatment, in early sessions, we engage in motivational interviewing with the child to help articulate the “pros” and “cons” of changing their behavioral responses. Children are often able to articulate what they do and do not “like” about their anger.

If inhibitory control mediates the efficacy of this treatment, we expect that narrow measurements of inhibitory control on standardized behavioral tasks would: (1) improve over the course of treatment, (2) would extend to new tasks (e.g., far transfer) and (3) would be associated with a decrease in irritability symptoms. To test this, the suite of behavioral tasks would be administered prior to the active treatment, as well as after treatment. Novel tasks would also be administered following treatment. Exploratory work would further probe if baseline deficits in inhibitory control are associated with treatment response.

Our specific hypothesis is that performance on standardized behavioral tasks would be impaired in youth with irritability prior to exposure-based cognitive behavioral therapy. Following treatment, we would expect youth to improve on average on inhibitory control tasks, with variability in improvements due to a multitude of factors including treatment response, co-occurring difficulties, the magnitude of pre-existing inhibitory control deficits, age, child's and parent’s ability to learn and their motivation to engage. Critically, improvement in irritability would specifically be mediated by the degree of improvement on the behavioral task assessments. Further extension of this work could examine whether, in treatment of irritability, repetitive, computer-delivered training and behavioral intervention may target different, complementary aspects of dysfunctional inhibitory control, with adjunct cognitive training potentially augmenting clinical response.

## Summary and future work

We proposed that inhibitory control, the ability to suppress a dominant response, may be a relevant treatment target for irritability in youth. We reviewed the challenges associated with measuring inhibitory control, the difficulties around different treatment approaches, and the need for integrated, ecologically valid treatments. We discussed how exposure-based cognitive behavioral therapy with parent management training may target acute deficits in withholding a prepotent response and support parents in reinforcing more long-term, adaptive, goal-directed behavior. By repeatedly teaching irritable children in the moment how to tolerate frustration without exerting a maladaptive behavioral response and coaching parents on how to consistently reinforce children’s behavior over time, we postulate that inhibitory control can be taught and will exhibit far-transfer if exercised in numerous emotionally evocative environments. However, this has never been systematically tested. The extent to which improvements in inhibitory control mediate clinical outcomes must be systematically assessed, ideally in targeted studies examining behavioral deficits in youth with irritability pre- and post-treatment using a suite of standardized tasks. If deficits in inhibitory control change prior to, and as a function of, treatment response, this provides a first step towards a mechanistic understanding of a behavioral intervention.
